# Risk of further surgery on the same or opposite side and mortality after primary total hip arthroplasty: A multi-state analysis of 133,654 patients from the Swedish Hip Arthroplasty Register

**DOI:** 10.1080/17453674.2018.1475179

**Published:** 2018-05-23

**Authors:** Peter H J Cnudde, Szilard Nemes, Erik Bülow, A John Timperley, Sarah L Whitehouse, Johan Kärrholm, Ola Rolfson

**Affiliations:** 1 Swedish Hip Arthroplasty Register, Centre of Registers, Gothenburg, Sweden;; 2 Department of Orthopaedics, Institute of Clinical Sciences, Sahlgrenska Academy, University of Gothenburg, Gothenburg, Sweden;; 3 Department of Orthopaedics, Hywel Dda University Healthboard, Prince Philip Hospital, Llanelli, UK;; 4 Hip Unit, Princess Elizabeth Orthopaedic Centre, Royal Devon & Exeter Hospital, Exeter, UK;; 5 Queensland University of Technology, Brisbane, Queensland, Australia

## Abstract

Background and purpose — The hip-related timeline of patients following a total hip arthroplasty (THA) can vary. Ideally patients will live their life without need for further surgery; however, some will undergo replacement on the contralateral hip and/or reoperations. We analyzed the probability of mortality and further hip-related surgery on the same or contralateral hip.

Patients and methods — We performed a multi-state survival analysis on a prospectively followed cohort of 133,654 Swedish patients undergoing an elective THA between 1999 and 2012. The study used longitudinally collected information from the Swedish Hip Arthroplasty Register and administrative databases. The analysis considered the patients’ sex, age, prosthesis type, surgical approach, diagnosis, comorbidities, education, and civil status.

Results — During the study period patients were twice as likely to have their contralateral hip replaced than to die. However, with passing time, probabilities converged and for a patient who only had 1 non-revised THA at 10 years, there was an equal chance of receiving a second THA and dying (24%). It was 8 times more likely that the second hip would become operated with a primary THA than that the first hip would be revised. Multivariable regression analysis reinforced the influence of age at operation, sex, diagnosis, comorbidity, and socioeconomic status influencing state transition.

Interpretation — Multi-state analysis can provide a comprehensive model of further states and transition probabilities after an elective THA. Information regarding the lifetime risk for bilateral surgery, revision, and death can be of value when discussing the future possible outcomes with patients, in healthcare planning, and for the healthcare economy.

Most patients undergoing total hip arthroplasty (THA) have an uneventful and relatively pain-free future, whereas some patients will have further health-care encounters related to their hip joints. These contacts may be for revision of the ipsilateral hip. The hip might also be re-operated on for reasons not necessitating exchange or extraction of the implant or any of its parts. Some patients will need surgery on the opposite hip and may also undergo re-operation or revision surgery on the second hip. Better knowledge of patients’ hip-related timeline (HRT) may improve the understanding and expectations of patients, surgeons, and healthcare providers. Further healthcare contacts are important in the case of bundled payments, tariffs, and payments-by-results (Burwell 2015, Jubelt et al. 2017). The increasing demands and financial pressures on health systems make predictions of further contacts with healthcare providers important.

Several studies have described the lifetime risk for revision and long-term mortality, but few have described the different paths (i.e., contralateral THA, revisions, and death) the patient can follow (Gillam et al. 2012, 2013, Abdel et al. 2016, Maradit Kremers et al. 2016, Sanders et al. 2017). The increased availability and quality of longitudinal data have stimulated the development of life-history models. Multi-state analysis has been advocated as a natural framework, studying transitions between different stages (Commenges 1999) and has been shown to provide a convenient framework for the handling of a wide variety of medical conditions, characterized by multiple events where longitudinal data are available (Farewell and Tom 2014). This framework allows for the combining of several possible outcomes in a single analysis and aids the depiction of the hip-related timeline that patients potentially could follow. This is in contrast with the more classical survival analysis, as employed in most studies, which is only able to depict transition from one stage to another. Additionally, multi-state analysis facilitates systematic handling of the laterality problem, an inevitably characteristic of every survival analysis in the field of arthroplasty (Ranstam et al. 2011).

We describe the probability for further hip-related surgery on the same or opposite side using the first primary total hip replacement on either side as the index operation and the probability of dying during the study period. We also depict the influences of known patient-related, surgery-related, and socio-economic factors using prospectively collected and linked data from a national database.

## Patients and methods

**Figure 1.**
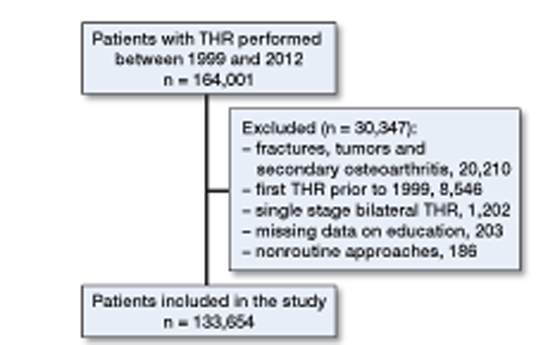
Flowchart of the patients included in this study (January 1, 1999–December 31, 2012).

Prospectively collected data from the Swedish Hip Arthroplasty Register (SHAR) were obtained and analyzed for all patients who received a first recorded primary THA for elective reasons (non-acute trauma-related and no tumor surgery) using the validated and linked research database to access surgical-, patient-related, and socio-economic factors (Cnudde et al. 2016). Data were available for 133,654 patients who underwent a first THA between January 1, 1999 and December 31, 2012 (Figure 1). The choice of study period was guided by the availability of the linked dataset for this period. All patients who had received a THA prior to this period were excluded. Bilateral single-stage primaries were also excluded (1,202 cases). The selected cohort of patients was then followed until the end of the study period (December 31, 2012) or death and revisions were recorded. We defined revision as exchange or extraction of the implant or any of its parts. The Swedish healthcare system provides universal access to healthcare for its residents and each hospital contact for every individual is recorded in a centrally governed system. Government databases also hold information on socioeconomic factors of all residents. Death dates are recorded by the Tax Office and are linked on a regular basis with the SHAR as well as other governmental databases. The SHAR is part of the Quality Registers in Sweden (QR) and the centralized information collection system in the Nordic Countries has been well recognized for its population-based research (Emilsson et al. 2015).

### Statistics

Continuous variables were summarized as means (SD), categorical variables as percentages.

**Figure 2.**
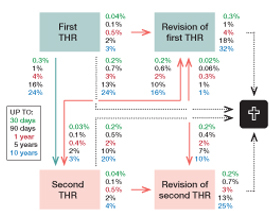
Multi-state analysis scheme and possible transitions. State 1 is the first hip replacement, state 2 is the second (contralateral) hip replacement, state 3 is the state where the first-performed hip is revised, whereas state 4 is the state where the contralateral hip is revised, and state 5 is death. The percentages stated are up to the 30- and 90-day mark as well as up to the 1-, 5-, and 10-year mark and represent the estimated transition probabilities within that given time.

To describe the association between ipsilateral operations, revisions, and mortality we adopted an extended irreversible disease progression illness–death model describing the movement of patients between a series of discrete states in a continuous time. This irreversible disease progression model had 5 discrete stages and describes the pathway of a patient from the first THA to the absorbing state of death as a Markov process. The patients enter the study at the time of their first elective THA surgery (State 1). They could stay in this state (unilateral THA without further intervention or death) until censoring on December 31, 2012—the end of the study period. However, the patient might advance into adjacent stages (Figure 2). If the contralateral hip has an arthroplasty the patient enters State 2, if the ipsilateral hip is revised then State 3 is applicable. A patient could reach the absorbing state, death (State 5). From State 2 the patients could advance to State 3 or State 4, which is revision of the second hip, or enter the absorbing State 5.

At any given time a patient can be in a specific state and the next state to which the patient moves and the time when this transition occurs is determined by a set of transition intensities that represents instantaneous risk of moving from state i to state j, namely:

$$q_{ij}=\;\begin{array}{c} \text{lim}\\ \delta t\to 0 \end{array}P(X(t+\delta t)\;=j|X\left(t\right)\;=i)/\delta t.$$

The transition intensities for one specified state to all others sum to 1, thus the diagonal elements of the transition intensities matrix Q that represent lingering in the specified state are given by *q_ii_* = –Σ*_i_*_≠_*_j_q_ij_*. We defined the transition ratio as the ratio of 2 estimated intensities (e.g. *q_ij_ ⁄q_ik_*). This ratio provides estimates on the likelihood of progressing to one stage or other. Statistical inference is based on approximate-normality and the δ-method. If the 95% confidence interval (CI) covers 1 we cannot reject the null-hypothesis of equal transition intensities.

We assessed the association between covariates x (sex, age, clinical diagnosis at first operation, comorbidity, surgical factors, socioeconomic status) and transition intensities with the modified proportional hazards model (Marshall and Jones 1995) calculated as

$$q_{ij}\left(x\left(t\right)\right)=q_{ij}{}^{\left(0\right)}exp(\beta_{ij}{}^{T}x\left(t\right)),$$

where *exp*(β*_ij_*) represents the estimated hazard ratios corresponding to given covariates effect of the transition intensities from state *i* to state *j*. Separate baseline hazards and regression coefficients are estimated for each possible transition by fitting a series of proportional hazards models (Andersen and Keiding 2002).

The conditional transition probabilities matrix *P(t)* is calculated as *e^tQ^* and its entries *p_ij_ (t)* are the probabilities of being in state *j* at time *t + u* given that at time *t* the patient is in state *i*.

The last estimates of interest are the unconditional state occupation probabilities, i.e., at each time point the curve estimates the fraction of patients currently in that state without considering the path that led there. This estimate is based on cause-specific hazard, *h(t_k_)* = (Σ*_i_ d_ki_*) ⁄*n_k_* where *d_ki_* is an indicator which takes value 1 if the patient transits to state *i* at time *t*, 0 otherwise and *n_k_* gives the number of patients at risk at time *t*. The unconditional state occupation probabilities are given by Σ*_ti_*_≤_*_t_ S(t_i–_*_1_*) h_k_ (t_i_)* where *S(t)* is the overall survival function, which summarizes the absorbing state (or censoring).

Statistical analyses were conducted with R computing environment (R Foundation for Statistical Computing, Vienna, Austria. https://www.R-project.org) and the “msm” package (Jackson 2011). 

### Ethics, funding, and potential conflict of interest

Ethical review approval was obtained on April 7, 2014 from the Regional Ethical Review Board in Gothenburg, Sweden (entry number 271-14).

This research received no specific grant from any funding agency in the public, commercial, or not-for-profit sectors. There was no support from any external organization for the submitted work and there are no financial relationships with any organizations that might have a direct interest in the submitted work in the previous 3 years.

## Results

Data were included on 160,165 primary THAs in 133,654 patients. During the study period 22,070 patients died, 26,511 patients had their contralateral hip replaced (simultaneous bilateral THAs have been excluded), 4,025 had their first replaced hip revised, and 694 their second hip. Patient demographics are presented in Table 1.

**Table 1.** Patient demographics of the cohort 1999–2012 (n = 133,654)

Age, mean (SD)	68 (11)
Sex, n (%)	
Male	57,058 (43)
Female	76,596 (57)
Diagnosis, n (%)	
Primary osteoarthritis	122,568 (92)
Inflammatory joint disease	3,199 (2.4)
Sequel childhood hip disorder	3,148 (2.4)
Femoral head necrosis	4,735 (3.5)
Elixhauser Index, mean (SD)	0.61 (0.96)
Surgical approach, n (%)	
Lateral	59,355 (44)
Posterior	74,299 (56)
Fixation, n (%)	
Cemented	104,560 (78)
Uncemented	13,500 (10)
Hybrid	3,336 (2.5)
Reversed hybrid	9,981 (7.5)
Resurfacing	1,666 (1.2)
Clinic type, n (%)	
University	14,080 (11)
County	44,897 (34)
Rural	55,126 (41)
Private	19,551 (15)

The median follow-up time from the first THA until death or censoring was 5.6 years, and from the second THA until death or censoring 4.2 years (Appendix 1, see Supplementary data).

**Figure 3.**
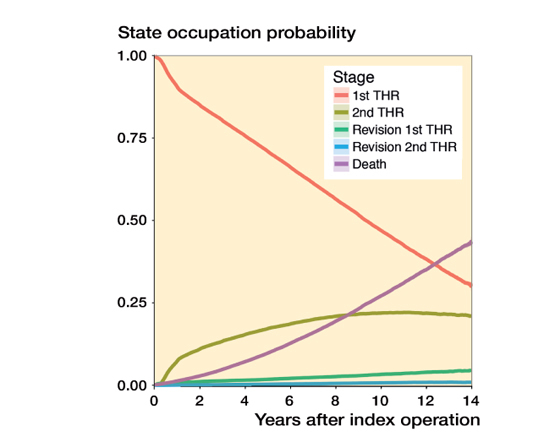
State occupation probabilities at different time points.

Transition probabilities (Appendix 2, see Supplementary data) between the different stages varied with time (Figure 2). Likewise, so did the probability of belonging to different stages (Figure 3). During the study period patients were twice as likely (transition ratio =2.1, CI 2.1–2.2) to have their contralateral hip replaced than to die. However, with passing time, probabilities converged and for a patient who remained in State 1 (1st THA) up to 10 years after the first hip surgery there was an equal chance of receiving a contralateral hip and dying (24%). Replacement of the contralateral hip was 7.5 times (CI 7.3–7.9) more likely than revision of the first hip. For patients who had their contralateral hip replaced, death as the next stage was 4.0 times (CI 2.4–6.6) more likely than revision of the first hip and 2.7 times (CI 1.7–4.5) more likely than revision of the second hip. For patients in state 2 (both hips sequentially replaced) the likelihood of revision of the second hip as a next step was 1.5 (CI 1.3–1.7) more likely than revision of the first hip. Viewed over the total period of observation the likelihood of revision of the first hip was, however, equal to the revision of the second hip (0.9, CI 0.8–1.0).

Hazard ratios of the most frequent transitions between states are provided (Table 2). They are also visualized in Forest plots (Appendix 3, see Supplementary data).

**Table 2.** Hazard ratios (95% confidence intervals) and the influence of different variables on the transition between different states ^a^

	Female	Lateral	Elixhauser	Education		
	sex	Age	approach	Comorbidity Index	Middle	High
State 1—State 2	1.17 (1.14–1.20)	0.98 (0.98–0.98)	0.94 (0.91–0.97)	1.04 (1.02–1.05)	1.09 (1.05–1.12)	1.18 (1.14–1.22)
State 1—State 3	0.71 (0.66–0.77)	0.99 (0.98–0.99)	0.98 (0.91–1.05)	1.18 (1.14–1.22)	1.06 (0.98–1.14)	1.08 (0.98–1.18)
State 1—State 5	0.65 (0.63–0.68)	1.09 (1.09–1.09)	1.01 (0.98–1.05)	1.19 (1.17–1.21)	0.86 (0.83–0.89)	0.78 (0.75–0.82)
State 3—State 5	0.68 (0.57–0.82)	1.08 (1.07–1.09)	0.83 (0.68–1.00)	1.25 (1.16–1.35)	0.99 (0.82–1.20)	0.83 (0.64–1.07)
Fixation:	Uncemented	Hybrid	Reverse hybrid	Resurfacing		
State 1—State 2	0.98 (0.93–1.03)	0.90 (0.84–0.97)	1.07 (1.01–1.13)	0.79 (0.71–0.88)		
State 1—State 3	1.15 (1.02–1.31)	1.08 (0.90–1.30)	1.34 (1.17–1.54)	1.60 (1.26–2.04)		
State 1—State 5	0.57 (0.51–0.64)	0.90 (0.80–1.02)	0.59 (0.52–0.66)	0.36 (0.22–0.60)		
State 3—State 5	0.87 (0.51–1.48)	1.25 (0.72–2.17)	0.78 (0.46–1.32)	0.48 (0.07–3.52)		

a State 1 = first THR; State 2 = second THR (contralateral); State 3 = revision of first THR (first operated side);

State 4 = revision of second THR (second operated side); State 5 = death.

**Figure 4.**
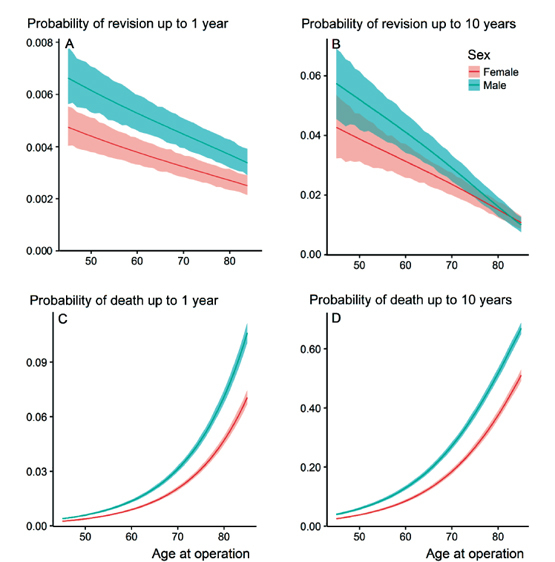
Effect of sex (male versus female) on transition probability from the state of 1st THR to revision of the first hip or death within 1 and 10 years from the index operation at different ages, presented with CI. A: Effect of sex on revision probability within 1 year. B: Effect of sex on revision probability within 10 years. C: Effect of sex on death probability within 1 year. D: Effect of sex on death probability within 10 years.

**Figure 5.**
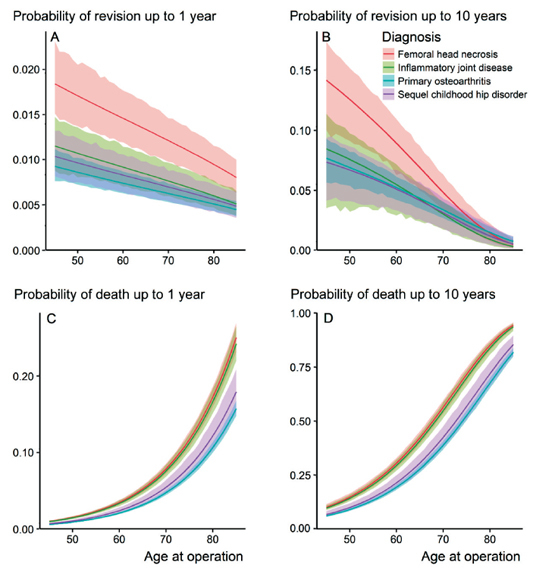
Effect of diagnosis (indication for surgery for the first hip) on transition probability from the state of 1st THR to revision of the first hip or death within 1 and 10 years from the index operation at different ages, presented with CI. A: Effect of diagnosis on revision probability within 1 year. B: Effect of diagnosis on revision probability within 10 years. C: Effect of diagnosis on death probability within 1 year. D: Effect of diagnosis on death probability within 10 years.

**Figure 6:**
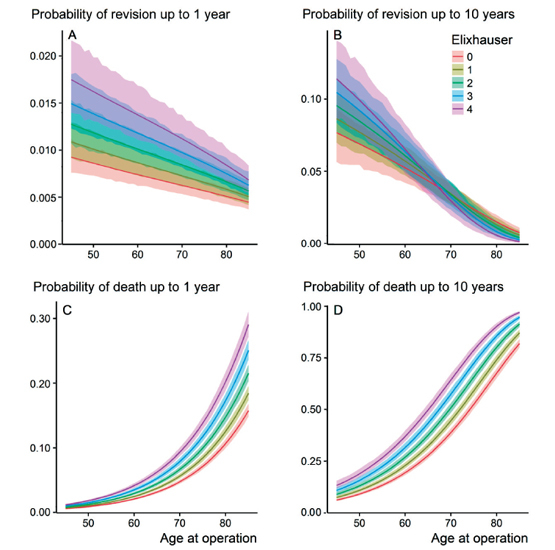
Effect of the Elixhauser Comorbidity Index (ECI) on transition probability from the sate of 1st THR to revision of the first hip or death within 1 and 10 years from the index operation at different ages, presented with CI. A: Effect of ECI on revision probability within 1 year. B: Effect of ECI on revision probability within 10 years. C: Effect of ECI on death probability within 1 year. D: Effect of ECI on death probability within 10 years.

Female patients were more likely to undergo surgery on both hips, but had less of risk of dying or being revised following surgery (Figure 4). Inflammatory joint disease as the indication for arthroplasty increased the likelihood of revision and dying and this influence was both age and time dependent. Operations performed for avascular necrosis of the femoral head were less likely to be performed on both sides and the risk of revision and/or dying was increased. Childhood hip diseases did not change the pattern of transitions if compared with primary osteoarthritis (OA) (Figure 5). The Elixhauser comorbidity index (compiled from the available ICD-10 codes in the year preceding surgery) had an effect on the probability of revision and an even greater effect on mortality (Figure 6). The majority of hip replacements were performed using posterior (56%) and lateral approaches (44%). We were unable to identify any significant effect of surgical approach on mortality or revision in the short or longer term (Appendix 4/1, see Supplementary data). The effect of fixation on revision at 1 year showed no statistically significant difference whereas at 10 years the effect gained significance from the age of 70 onwards. The difference in mortality could be identified at 1 and 10 years (Appendix 4/2, see Supplementary data). Patients who had obtained only a lower education level were less likely to undergo sequential bilateral procedures and showed an increased likelihood of dying (Appendix 4/3, see Supplementary data).

## Discussion

The multi-state analysis enabled the comprehensive prediction of transition probabilities between different postoperative states and the influence of patient demographics, patient- and surgery-related factors as well as socioeconomic influences. Using longitudinally and prospectively collected data from nationwide registers we could describe part of the patient’s journey following the first hip replacement for elective reasons (the hip-related timeline). Multi-state analysis has the advantage of providing a better understanding of the data and a more coherent picture of the complete path instead of isolated events (Gillam et al. 2012).

The number of patients with sequential bilateral THA is increasing, as also seen during our study period (Cnudde et al. 2018a). Describing the factors contributing to the contralateral operation is of interest. The probability of undergoing a further hip replacement on the contralateral side was 1 in 4. A previous register study also used this technique but in a smaller cohort and with a shorter study period Gillam et al. (2012, 2013) evaluated the risk of subsequent contralateral THA using a multi-state analysis and found a 16% and 20% probability of receiving a contralateral hip in the Australian and Norwegian population respectively. Shao et al. (2013) described a 31% chance of receiving a contralateral THA at a mean of 18 years after original surgery. These figures are similar to what we found in Sweden. As expected the risk of revision decreases with increasing age, as a result of selection and age as a competing risk. The lifetime risk for revision has been previously studied and the age at the time of the operation has been found important (Abdel et al. 2016, Bayliss et al. 2017, Schreurs and Hannink 2017). Compared with an age-matched population, mortality remains somewhat lower in the THA population, more so in the older age groups (Cnudde et al. 2018b).

The influence of diagnosis on mortality has been described previously, with patients undergoing THA for primary OA doing better than other diagnoses (Lie et al. 2000, Pedersen et al. 2011, Cnudde et al 2018b). The influence of diagnosis on revision surgery after adjusting for co-variables is obvious, with increased revision rates in the case of inflammatory arthritis or avascular necrosis of the femoral head. Bergh et al. (2014) used the Nordic Arthroplasty Register Association (NARA) database to study the effect of avascular necrosis on revision rates and found an increased revision rate in this group compared withc primary OA. This increased revision rate was not confirmed in a systematic literature review by Johannson et al. (2011). Register studies and prospective studies also describe higher dislocation rates and an increased risk of periprosthetic fractures in patients with inflammatory arthritis undergoing THA (Zwartele et al. 2004, Lindahl et al. 2006, Meek et al. 2008). This will have a bearing when comparing outcomes between hospitals and surgeons with a different case-mix. However, our data do not include reoperations not necessitating exchange or extraction of the implant or any of its parts (e.g., fixation of periprosthetic fractures or reduction of dislocated implant).

The importance of comorbidity on parts of the hip-related timeline, and especially on mortality, has been studied extensively. Even if the predictive power of this factor has been considered weak, the importance of comorbidity cannot be ignored (Glassou et al. 2014, 2017, Bulow et al. 2017). In view of the increasing length of survival following arthroplasty (Cnudde et al. 2018b) one has to consider that relevant comorbidity might develop and cause both morbidity (potentially leading to increased risk of infection and periprosthetic fracture) and mortality. Hunt et al. (2017) have described malignancies, cardiovascular disease, and respiratory disease as the main causes of mortality following arthroplasty surgery.

In our study, patients who belonged to less educated groups were less likely to progress to a second-side operation but were equally likely to undergo revision surgery and had a higher risk of dying. The association between socioeconomic factors and increased risk of dying has been described by Whitehouse et al. (2014) in the United Kingdom and by Bennett et al. (2010) in the USA.

Abdel et al. (2016) published the lifetime risk of revision using death as competing risk as well as Kaplan–Meier survivorship for a cohort of patients from their institution using the original Charnley cemented THA (DePuy International Ltd, Leeds, UK). We could identify the same effects of sex and age on both revision risk and death, using nationwide data and multiple implants as well as different fixation methods.

We believe our study adds more support to the question of what will happen with time following a patient’s first replacement hip. Females are in generally more likely to receive bilateral hip replacements during their lifetime than their male counterparts and at an earlier age. We have not studied the influence of type of fixation in detail because there are too many possible confounding factors to consider within the framework of this study. However, the effect of fixation on revision at 10 years is, in our belief, quite important and strengthens the guidance that purely for revision reasons a cemented implant would be the implant of choice for patients older than 70 years. The differences in mortality rate can well be explained by patient selection as the uncemented implants in Sweden are mainly used in patients with better bone quality and better mobility.

We cannot describe the effect of an anterior approach on the probability of transitions as during the study period this approach was used very rarely. Hunt et al. (2013) described improved early survival in patients operated with the posterior approach. We were unable to identify a statistically significant difference on most transitions with the exception of a minimal effect on bilateral procedures and decreased hazard ratio in transition from state 3 to 5 (revision of first hip to death). We were unable to show any statistically significant difference between approaches on mortality or revision rate. This gives further information for informed discussion on the choice of approach; the surgeon’s preference and possible differences in PROMs should, however, also be considered (Smith et al. 2012, Lindgren et al. 2014).

### Limitations and strengths

For this analysis, patients who had had more than 1 ipsilateral revision were considered as staying in the revision group (they will remain in the same group). Patients undergoing multiple surgeries on the same hip might well have attendant possibilities for morbidity and these are not visible within the timeline. The number of patients that are undergoing multiple revisions has been limited and the complexity of adding additional states would, in our view, not be beneficial for the model. We are aware of bias in the decision-making regarding when to perform second-sided surgery/revision surgery. It is possible that some patients—despite being in need for revision or a contralateral hip operation—might not be operated upon as a result of decisions by the surgical team. We are also aware that some operations such as revisions for infections, periprosthetic fractures, and dislocations are under-reported within many of the registers (Slobogean et al. 2015). Comorbidity may well be under-reported as comorbidity records depend heavily on careful recording of comorbidity also recorded at the secondary care (Bulow et al. 2017). However, the existing measures of comorbidities have limited value in the case of revisions or death of THA patients. We believe that with an increased follow-up time our series could give a more in-depth view into what happens in the longer term. In addition, an association has been described between monoarthrodial pathology in the hip, the progression of degenerative change in the contralateral knee, and subsequent requirement for knee arthroplasty (Shakoor et al. 2014). Further studies have described associations with spinal or knee replacement surgery as degenerative or inflammatory changes are seldom limited to a single joint (Gillam et al. 2012, 2013). Our study, however, has not linked the available information with the knee or spine registries.

The major strength of our study is the completeness and validity of the data within the register. Using the Swedish personal identity numbers it is unlikely we underreport mortality as every death is recorded by the tax office and subsequently in the SHAR database. A second strength is the longevity and the size of this register.

This prospective nationwide program, collecting data from multiple surgeons working in multiple institutions, was set up as a quality improvement tool but the strength and validity of the data can provide us with answers to many unsolved questions. Our results of this study contribute to a better understanding of the hip-related pathway patients are following after their initial surgery.

### Supplementary data

Appendices 1–4 are available as supplementary data in the online version of this article, http://dx.doi.org/10.1080/17453674.2018.1475179

Conceived the study: PC, SN, JT, OR, JK. Data collection: PC, SN. Statstical analysis: SN, EB, SW, JK. Drafting of the manuscript: PC, SN. All authors performed data analysis and editing of the manuscript.

The authors would like to acknowledge the hard work of the register coordinators, Kajsa Erikson, Karin Davidsson, Karin Lindborg, and Karin Pettersson at the SHAR and the orthopedic surgeons and coordinators at all contributing hospitals for providing them with high-quality data. The authors are also very grateful to Charlotte Vitse and Daniel Odin for their contributions to the graphical representation of the multi-state analysis.

*Acta* thanks Stein Atle Lie and Esa Jämsen for help with peer review of this study.

## Supplementary Material

IORT_A_1475179_SUPP.pdf
