# Supplementary material for: Risk of further surgery on the same or opposite side and mortality after primary total hip arthroplasty: A multi-state analysis of 133,654 patients from the Swedish Hip Arthroplasty Register
Source: Acta Orthop. 2018 May 23;89(4):386–93. doi: 10.1080/17453674.2018.1475179 (PMC6066773; doi:10.1080/17453674.2018.1475179)
Supplement: IORT_A_1475179_SUPP.pdf [file IORT_A_1475179_SM0638.pdf]

Supplementary data

Appendix 1. Follow-up and numbers at risk

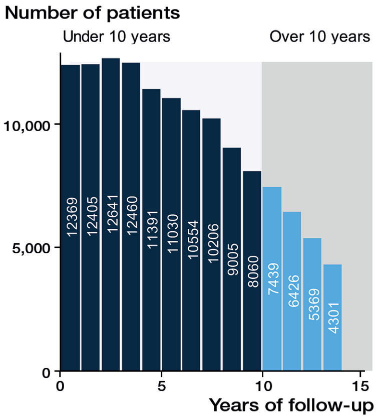

Figure 1. Number of patients and follow-up years.

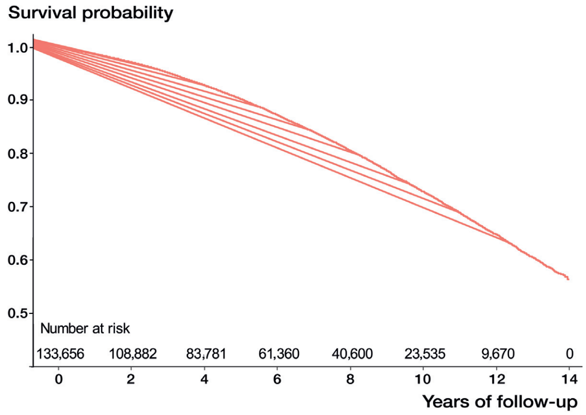

Figure 2. Survival probability and numbers at risk.

Appendix 2. Transition probabilities

Transition probability estimates within a given time period and associated 95 % confidence intervals based on the normality assumption.

	State 1	State 2	State 3	State 4	State 5
30 days					
State 1	0.9939 (0.9938–0.9939)	0.0033 (0.0032–0.0033)	0.0004 (0.0004–0.0005)		0.0024 (0.0023–0.0024)
State 2		0.9974(0.9973–0.9975)	0.0003 (0.0003–0.0004)	0.0004 (0.0004–0.0005)	0.0018 (0.0016–0.0018)
State 3		0.0021 (0.0019–0.0024)	0.9942 (0.9938–0.9946)	0.0002 (0.0001–0.0003)	0.0034 (0.0031–0.0037)
State 4			0.0015 (0.0010–0.0020)	0.9962 (0.9953–0.9969)	0.0023 (0.0017–0.0029)
State 5					1.0000 (1.0000–1.0000)
90 days					
State 1	0.9818 (0.9815–0.9819)	0.0098 (0.0097–0.0099)	0.0013 (0.0012–0.0013)		0.0071 (0.0070–0.0072)
State 2		0.9924 (0.9921–0.9926)	0.0010 (0.0009–0.0011)	0.0013 (0.0012–0.0014)	0.0053 (0.0050–0.0055)
State 3		0.0064 (0.0057–0.0070)	0.9828 (0.9816–0.9838)	0.0006 (0.0004–0.0009)	0.0102 (0.0094–0.0112)
State 4			0.0044 (0.0032–0.0061)	0.9888 (0.9986–0.9907)	0.0068 (0.0052–0.0087)
State 5					1.0000 (1.0000–1.0000)
1 year					
State 1	0.9280 (0.9274–0.9286)	0.0383 (0.0378–0.0388)	0.0050 (0.0048–0.0052)		0.0285 (0.0281–0.0289)
State 2		0.9694 (0.9684–0.9704)	0.0041 (0.0044;0.0044)	0.0052 (0.0048–0.0056)	0.0212 (0.0204–0.0220)
State 3		0.0248 (0.0222–0.0276)	0.9321 (0.9275–0.9360)	0.0025 (0.0018–0.0035)	0.0406 (0.0374–0.0439)
State 4			0.0172 (0.0122–0.0233)	0.9553 (0.9453–0.9631)	0.0273 (0.0215–0.0353)
State 5					1.0000 (1.0000–1.0000)
5 yearS					
State 1	0.6880 (0.6859–0.6904)	0.1563 (0.1547–0.1582)	0.0202 (0.0195–0.0208)		0.1327 (0.1311–0.1345)
State 2		0.8571 (0.8527–0.8617)	0.0174 (0.0161–0.0190)	0.0225 (0.0208–0.0242)	0.1027 (0.0989–0.1066)
State 3		0.1015 (0.0917–0.1118)	0.7046 (0.6875–0.7195)	0.0110 (0.0081–0.0149)	0.1827 (0.1704–0.1962)
State 4			0.0688 (0.0507–0.0893)	0.7966 (0.7566–0.8271)	0.1319 (0.1047–0.1609)
State 5					1.0000 (1.0000–1.0000)
10 years					
State 1	0.4738 (0.4707–0.47693)	0.2437 (0.2409–0.2463)	0.0311 (0.0298–0.0322)		0.2443 (0.2414–0.2471)
State 2		0.7366 (0.7297–0.7438)	0.0288 (0.0263–0.0314)	0.0375 (0.0343–0.0403)	0.1970 (0.1908–0.2034)
State 3		0.1586 (0.1444–0.1750)	0.4991 (0.4749–0.5221)	0.0189 (0.0141–0.0250)	0.3234 (0.3030–0.3447)
State 4			0.1115 (0.0779–0.1333)	0.6346 (0.5723–0.6835)	0.2494 (0.2050–0.2983)
State 5					1.0000 (1.0000–1.0000)

Appendix 3. Hazard rates

Hazard ratios and associated 95% confidence intervals. The hazard ratios correspond to the covariate effect on the transition intensities.

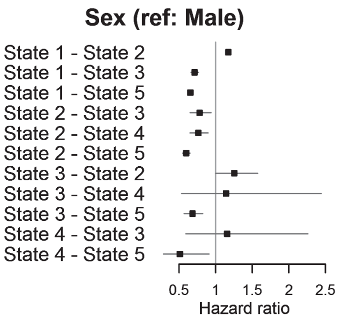

Figure 1. Effect of sex on the transition between different states.

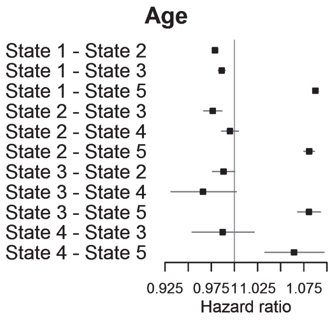

Figure 2. Effect of age on the transition between different states.

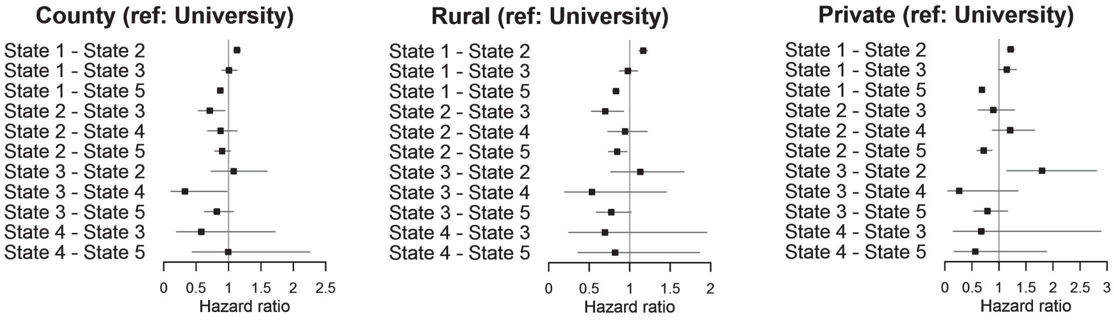

Figure 3. Effect of clinic type on the transition between different states.

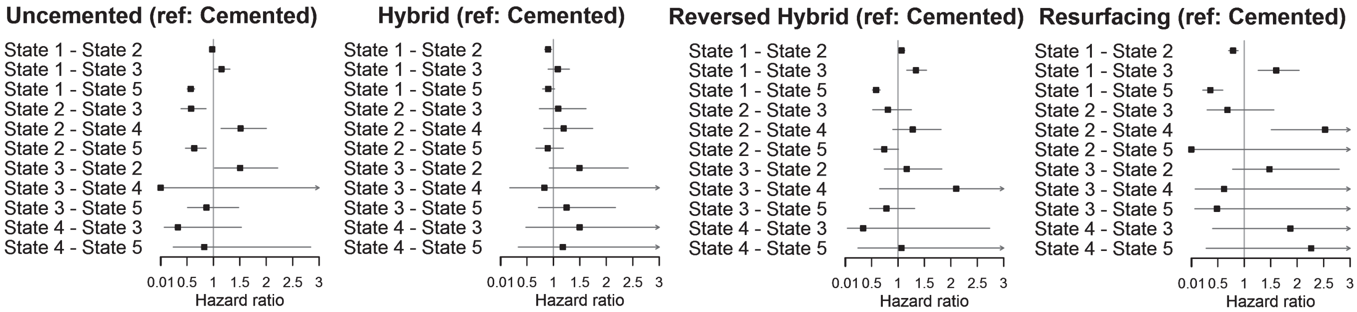

Figure 4. Effect of fixation on the transition between different states.

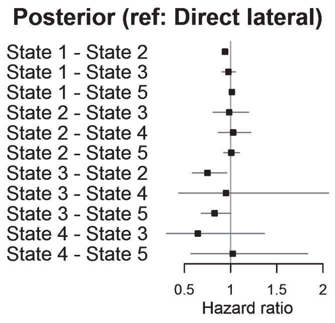

Figure 5. Effect of surgical approach the transition between different states.

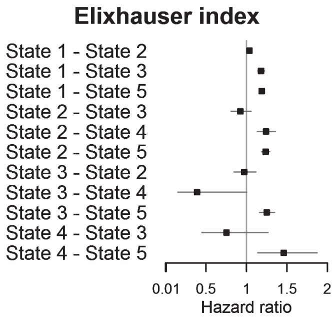

Figure 6. Effect of Elixhauser comorbidity index on the transition between different states

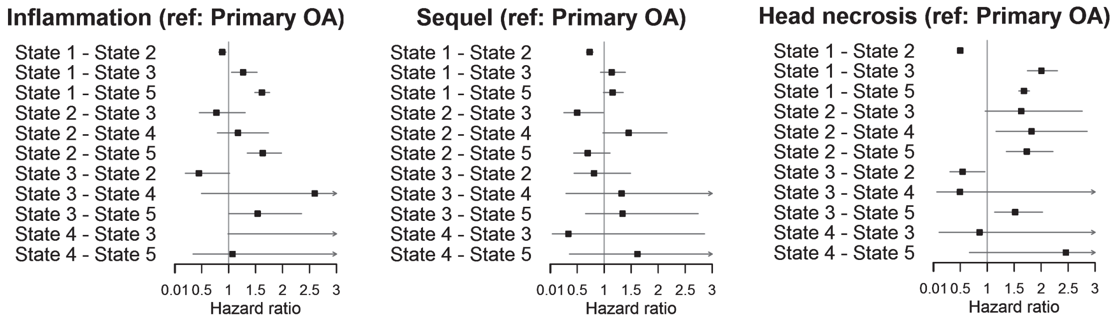

Figure 7. Effect of diagnosis on the transition between different states.

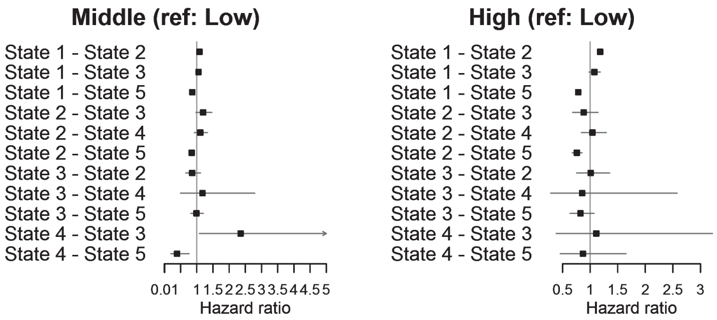

Figure 8. Effect of education the transition between different states.

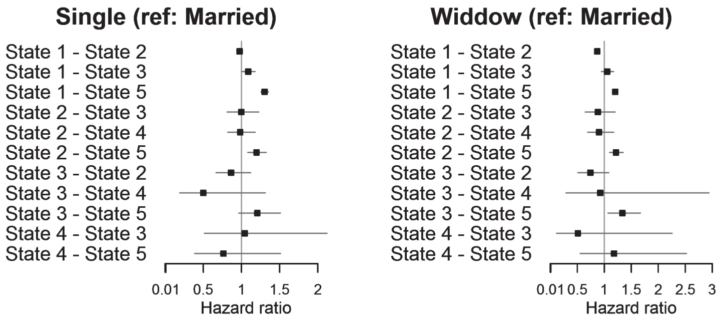

Figure 9. Effect of civil status on the transition between different states.

 Acta Orthopaedica 2018; 89 (DOI: 10.1080/17453674.2018.1475179)

Appendix 4. Transition probabilities

Age and covariate specific transition probabilities and associated 95 % confidence intervals.

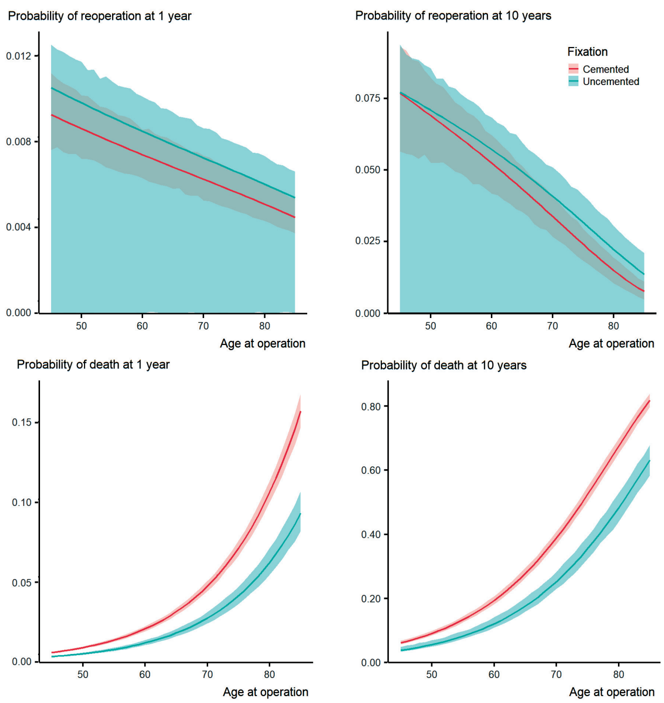

Figure 1. The effect of fixation on transition probability from the state of 1st THR to revision of the first hip or death up to 1 and 10 years from the index operation at different ages.

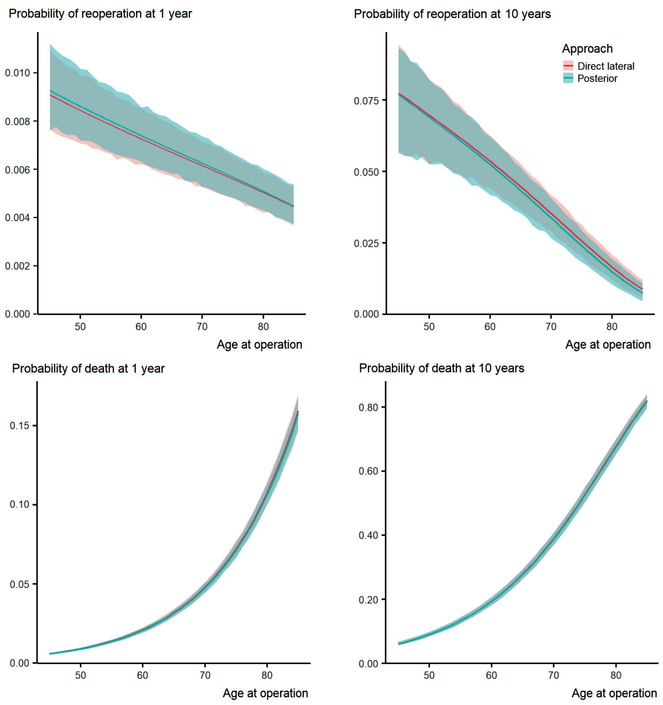

Figure 2. The effect of surgical approach on transition probability from the state of 1st THR to revision of the first hip or death up to 1 and 10 years from the index operation at different ages.

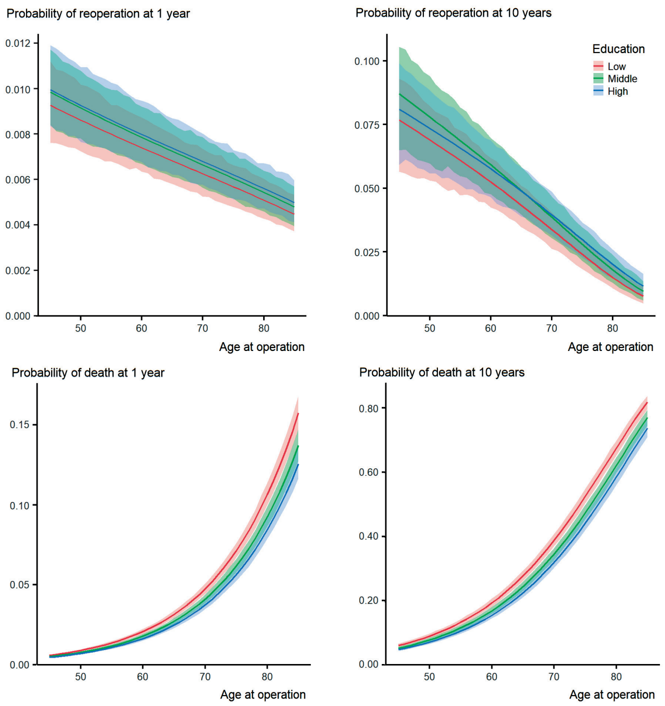

Figure 3. The effect of education level on transition probability from the state of 1st THR to revision of the first hip or death up to 1 and 10 years from the index operation at different ages.
